# Modelling the spread of American foulbrood in honeybees

**DOI:** 10.1098/rsif.2013.0650

**Published:** 2013-11-06

**Authors:** Samik Datta, James C. Bull, Giles E. Budge, Matt J. Keeling

**Affiliations:** 1 WIDER group, School of Life Sciences, University of Warwick, Coventry CV4 7AL, UK; 2 National Bee Unit, Food and Environment Research Agency, Sand Hutton, York YO41 1LZ, UK

**Keywords:** epidemiology, Bayesian, MCMC, likelihood, honeybee, American foulbrood

## Abstract

We investigate the spread of American foulbrood (AFB), a disease caused by the bacterium *Paenibacillus larvae*, that affects bees and can be extremely damaging to beehives. Our dataset comes from an inspection period carried out during an AFB epidemic of honeybee colonies on the island of Jersey during the summer of 2010. The data include the number of hives of honeybees, location and owner of honeybee apiaries across the island. We use a spatial SIR model with an underlying owner network to simulate the epidemic and characterize the epidemic using a Markov chain Monte Carlo (MCMC) scheme to determine model parameters and infection times (including undetected ‘occult’ infections). Likely methods of infection spread can be inferred from the analysis, with both distance- and owner-based transmissions being found to contribute to the spread of AFB. The results of the MCMC are corroborated by simulating the epidemic using a stochastic SIR model, resulting in aggregate levels of infection that are comparable to the data. We use this stochastic SIR model to simulate the impact of different control strategies on controlling the epidemic. It is found that earlier inspections result in smaller epidemics and a higher likelihood of AFB extinction.

## 1. Introduction

Globally, bees contribute immensely to agriculture through crop pollination. A recent report indicated that 71 out of 100 important crop species are bee-pollinated [1]. Honeybees (*Apis mellifera*) are a commercially important managed pollinator and the most common bee species in the world [2]. The impact of pollination by honeybees upon the global economy has therefore been estimated to be hundreds of billions of dollars [3,4].

In the past 20 years, there has been a marked increase in the level of disease in bee populations [5]. The Varroa parasite (*Varroa destructor*), along with a host of bacterial pathogens such as European foulbrood (EFB) and American foulbrood (AFB) [6,7], parasitic insects such as the small hive beetle [8–10] and *Tropilaelaps* mite [11] and viruses such as the Kashmir bee virus [9,10] and the Israeli acute paralysis virus [12], have all been implicated in honeybee colony loss. Such losses have led to reduced pollination leading to lower crop yields, such as almonds in California [13]. AFB has been found to be an unusually virulent pathogen with a high kill rate (see [14]).

In an effort to control disease spread between apiaries, a variety of strategies have been implemented in the past, with varying degrees of success. Different strategies are employed by the respective authorities in charge between countries. In England and Wales, for example, AFB is always treated by burning infected colonies to eradicate the disease [15]; by contrast, oxytetracycline (OTC) has been used in the USA since the 1950s, as an antibiotic for treating both AFB and EFB [16]. An alternative treatment is shook swarm; this involves the transfer of only the adult bees from diseased combs to fresh disease-free equipment, in order to separate the bees from the disease and avoid total colony destruction. This method has been considered to be comparable to the use of OTC in recent years [17–19]. As with any farmed species, the destruction of animals is always the last resort where all other measures are insufficient to halt the continued spread of the disease. An internationally accepted method for preventing disease spread between apiaries has yet to be reached.

The disease we investigate here is AFB, caused by the pathogenic bacterium *Paenibacillus larvae*, that affects only the larval stages of honeybees, by infecting them 12–36 h after hatching and spreading via spores after the death of the larvae [7,14]. The main mode of AFB transmission is horizontal, via honeybee behaviours such as robbing and the movement of infected honey stores, as well as indirect bee-to-bee contact such as contaminated water [14]. This paper deals with an AFB epidemic that took place during the summer of 2010 on the island of Jersey, a relatively small island (with an area of 46 square miles) situated off the northwest coast of France. All apiarists registered their hives with the States of Jersey, and thus information about the location and owner of all hives on the island was known (the wild population of honeybees is relatively small, and so a complete dataset was assumed). Visits were arranged to inspect all apiaries during June (with each apiary containing one or more hives), and repeat visits in August were made to apiaries found to contain AFB-positive hives at the first visit.

With the information provided in the dataset, we construct a robustly parametrized spatial SIR model with an underlying network of ownership. The aim of this model is to elucidate the main features of transmission and to understand the impact of alternative control strategies. A rigorous Bayesian Markov chain Monte Carlo (MCMC) methodology (using exact likelihoods) is developed to infer distributions of parameter constants in the model, as well as the infection time of all AFB-positive hives in the dataset. Similar MCMC methods have been used to describe livestock diseases [20,21], but not to our knowledge applied to epidemics in honeybee populations until now. Our system is also complicated by the lack of owner reporting and the sparsity of the inspection data. The results of the analysis are tested by using parameter values from the MCMC in a stochastic SIR model, and we compare predicted levels of infection in June and August to those from the data. Finally, a suite of simulated control strategies are implemented to compare plausible methods for eliminating or limiting the spread of future AFB outbreaks.

## 2. Data and methods

### 2.1. Data collection

The dataset was acquired by an initial census carried out between 1 and 18 June 2010, following a report of suspected EFB on 31 May 2010, which was confirmed to be AFB 3 days later. Follow-up inspections were carried out of infected apiaries between 8 and 16 August; some follow-up inspections were also carried out even if the apiary was AFB-negative. In total, 199 visits were carried out on 130 different apiaries, with a total of 458 hives being examined for AFB. The data collected from the survey comprised the following information: colony reference (a unique identifier for each apiary on the island, so repeat visits can be identified), owner reference (a unique label for each owner, who may own one or multiple apiaries on Jersey), number of honeybee colonies at each apiary (this occasionally changed between inspections, owing to hive addition or removal), *x*- and *y*- coordinates, number of AFB-positive hives in the apiary and the date of inspection. Whenever an inspection was carried out, if AFB was presented in the hive, then the hive was destroyed and the parts scorched, to guarantee removal of the disease. Thus, after reporting an infection, the hive can no longer transmit infection to other hives.

Although information about the number of combs of brood and bees was available for some inspections, it was not complete, so we choose not to use the apiary-specific data; instead, we assume hives are homogeneous with equal susceptibility and infectiousness.

### 2.2. Model formulation

We capture the dataset using an SIR model (standing for, respectively: susceptible, infected, removed). We introduce the vectors **S**, **I** and **R** to denote, respectively, the creation, infection and removal times of all hives. For inspections where AFB is not detected, the time of the negative inspection is recorded in an additional vector **R**^−^. We label the number of hives *n*, and the date of the last inspection *T*. Our model adds to the complexity of the classic SIR model by way of spatial interactions, a network of ownership and stochasticity in the spread of infection.

Diseases such as foot-and-mouth involve authorities following up on alerts from farmers [22,23], in which case a relationship can be assumed between infection time and detection time. This is not the case with AFB, which can be hard to identify by sight in the hive by beekeepers; several reports from farmers received in 2010, who were suspicious of infected hives, were both revealed to be free of AFB. The initial inspections were carried out on all hives indiscriminately as a census; in this sense, AFB is similar to bovine tuberculosis [24,25] in that it is difficult to detect by farmers. We use the removal times from the data to estimate infection times which are, as is often the case with epidemiological data, unknown.

We allow for a time period where a hive is infected but not yet infectious; we call this time the *latent period*, which we denote as *l*(*t*). In theory, any function that increases from 0 to 1 could be used, such as a step function; for our model, we choose a more biologically realistic function,2.1
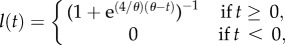
where 4/**θ** determines the steepness of the switching function and **θ** determines the time where the switch from infected to infectious occurs (so as the latency period increases, the switch from 0 to 1 becomes more gradual).

The disease transmission rate between an infected hive *i* and susceptible hive *j* is constructed as2.2
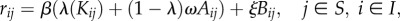
with2.3
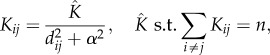
where **β** is the overall rate multiplier for the infection rate from infectious hives to susceptible ones; **λ** is the proportion of the infection spread due to distance, as opposed to ownership (0 ≤ **λ** ≤ 1); **ω** scales the amount of infection spread by the owner (constant and independent of apiary size), while *A_ij_* = 1 if hives *i* and *j* have the same owner and is zero otherwise; ξ*_ij_* is additional apiary-specific infectious pressure, while *B_ij_* = 1 if hives *i* and *j* are on the same apiary and is zero otherwise; **α** is the distance exponent, which controls how quickly infectiousness drops off as the distance between hives increases (smaller values of **α** cause a more rapid decline in distance-related transmission); *d_ij_* is the Euclidean distance in kilometres between hives *i* and *j* (*d* = 0 for hives on the same apiary) and *n* is the number of hives present on the entire island.

Thus, the total rate of infectious pressure upon a susceptible apiary *j* at time *t* is2.4
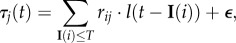
where **ε** is a constant background infection rate, unrelated to the infection status of all other hives. This is to account for other sources of infection not explicitly covered in the model, such as immigration of the disease from abroad and also improves the likelihood calculated in the MCMC scheme.

As all suspected infections were immediately confirmed in the field using test kits for AFB (Vita Europe Ltd) similar to those reported for EFB [26], we assume that the data are accurate, with no false positives. However, as the disease takes time to become symptomatic in the hive, we assume that infections may exist, which were not present in the data. This may be either because of the inspection being too soon after the infection reached a particular hive, or because of infection spreading to the hive between the last inspection and the end of the inspection period. For the former case, we introduce a detection probability function independent of the latency of the disease (2.1), as the ability to detect AFB in the hive may not correlate directly with infectivity. Thus, the probability of a positive result given that the hive is infected with *P. larvae* is modelled as2.5
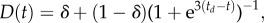
where **δ** > 0 ensures non-zero probabilities of detection for small values of *t*, and *t_d_* determines where the switch to almost guaranteed detection occurs. Information about the detectability of the disease was acquired from contacts on Jersey and at FERA, and from this we set *t_d_* = 10 days. Undetected infections in colonies are labelled as *occults*, following previous work [20,21]. The MCMC scheme in §2.3 is used to determine the number of occult infections (if any), which may exist within the dataset.

### 2.3. MCMC scheme

We set up a statistical model for analysing our epidemic data, based on techniques developed by O'Neill & Roberts [27], designed to analyse spatial epidemic data using Bayesian MCMC methodology (an applied example is modelling foot-and-mouth disease in cattle, see [20,21]). The basic premise involves: setting the model up an initial parameter set **Ω** (i.e. both values for the model constants and infection times for AFB-positive hives), calculating the initial likelihood, and then with each iteration altering one of the parameters, recalculating the likelihood and choosing whether to accept the new set of parameters based on a comparison of the likelihoods (for more information in acceptance, see appendix A.3). This MCMC algorithm efficiently explores the whole parameter space, and the sets of parameters accepted define the (posterior) distributions taking fully into account all uncertainties in the data.

The likelihood is calculated as follows:2.6
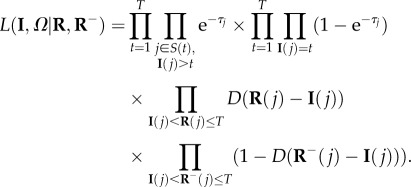


The four products are, respectively, the probabilities of

(1) remaining susceptible while under infectious pressure from other hives,
(2) becoming infected on day *t*,
(3) AFB being detected at an inspection, where the hive is diseased, and
(4) AFB not being detected at an inspection, where the hive is infected but not yet symptomatic.

Prior distributions for all parameters are required to carry out the MCMC scheme. As no previous analyses have been carried out on disease spread in honeybee populations that we are aware of, information about likely parameter values is difficult to find. Thus, gamma distributions were used as priors for all parameter constants in the model, except where upper limits could be imposed, in which case beta distributions were used (for more information see appendix A.2).

The timescale that we choose to work on is from 1 January 2009 (to account for the possibility that AFB was present from the previous year) until the last inspection date, 16 August 2010. Sources from both the NBU and local bee inspectors informed us that little to no beekeeping activity generally occurs outside the March–October period. To account for this, we assume that no AFB is spread between hives outside the beekeeping season. To this end, we allocate a four-month ‘freeze’ period over the 2009/2010 winter (1 November 2009–28 February 2010), during which no disease transmission occurs.

### 2.4. Stochastic susceptible, infected, removed model

To confirm that the results from the MCMC are reliable, we construct a spatial SIR model, using the coordinates and owner network from the dataset, with which to test the output from the MCMC scheme. For each simulation, we require values for the parameter constants, as well as the initial infection time and hive. For each run of the SIR model, we randomly sample a set of parameters from 10^4^ saved outputs from the MCMC scheme, and allow the model to run until the end of the inspection period. The primary inspections from the data are used, and wherever an infection is found during a primary inspection, the hive is removed and a follow-up inspection is carried out on a random day within the August period (8–16 August 2010). This is in keeping with the strategies involved during the epidemic on Jersey. The validity of the model formulation and parameters is tested by comparing the predicted total number of detected infections in the two censuses to the data; this provides a test that is largely independent of the fitting procedure. (In appendix A.5, we also show receiver operating characteristic (ROC) curves that provide an additional level of validation.)

We then simulate different control strategies and observe the consequences they have on the spread of AFB. Control methods are relatively simple to simulate and can provide invaluable insights into their potential to limit the spread of infection. The inspections carried out in Jersey involved destruction of any diseased hives immediately upon detection of AFB. We also test the effects of: performing a complete census with the follow-up inspections, carrying out secondary inspections of any apiaries within a fixed radius of any infected hives [22,28], carrying out secondary inspections on any hives owned by the same owners, and carrying out the initial and follow-up inspections earlier in the year than the original June and August 2010 (respectively). We also test combinations of these strategies to find an optimum strategy.

For a detailed breakdown for the set-up and components which make up the MCMC scheme, along with the resulting plots, see appendix A.5.; in §3, we present the main findings of our analysis of the Jersey data.

## 3. Results

### 3.1. Model constants

Figure 6 shows the results of running the MCMC scheme (see §2.3 for an outline, and §5 for a detailed breakdown of the methods and complete analysis), to determine credible values of the model constants. The constants are taken from equations (2.2) to (2.5), with descriptions given under the respective equations.

The MCMC chain is well mixed and appears to explore the parameter space thoroughly. The model constants are well defined, with Gaussian-shaped histograms. The scheme was initialized at a variety of regions of parameter space to test the convergence, and similar values for both the model constants and the likelihood were consistently observed.

### 3.2. Characteristics of the epidemic

**Figure 1.**
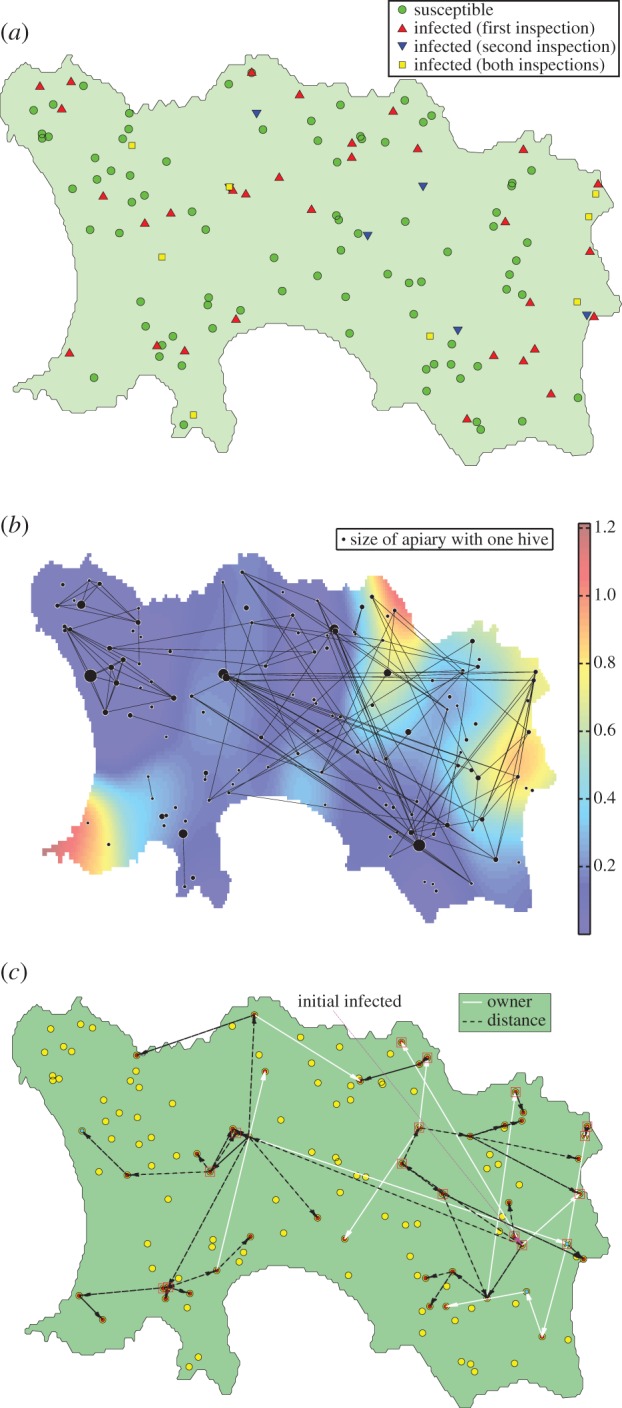
Summary of results from the MCMC. All plots show the island of Jersey with characteristics of the observed epidemic overlaid. (*a*) The infection status of apiaries by the end of the 2010 inspection period. The four categories are susceptible, infected (first inspection), infected (second inspection) and infected (both inspections). (*b*) The apiaries present on Jersey during the 2010 epidemic, scaled by the number of hives present during the epidemic. Overlaid are the ownership network (black lines) and the likelihood map for the location of the primary infection during the AFB outbreak (see colour scale). (*c*) An example of a typical infection map obtained from the MCMC scheme (i.e. a random iteration selected from the scheme). Uninfected apiaries are in yellow, apiaries containing one or more infected hives are in red, apiaries containing occult hives are in blue. Arrows show the probable source of infection for each hive; solid white arrows indicate transmission by the owner, dashed black arrows indicated distance-based transmission. The initial infection is highlighted.

Using the MCMC scheme, we are able to ascertain various information about the data from the observed epidemic. The results are summarized in figure 1.

As shown in figure 1, most apiaries were AFB-negative, with 46 out of 130 being classed as infected during the inspection period. Primary cases of AFB appear to be scattered across the island, although most cases tended to be in the Eastern area and across the north; the south and southeast regions of the island were relatively AFB-free.

Figure 1*b* shows the size of the apiaries on Jersey (a larger number of hives is indicated by a larger radius). Overlaid is the owner network which connects apiaries owned by the same beekeeper. By repeatedly running the MCMC scheme, it is possible to plot the distribution of initial infections, to estimate where the origin of the AFB outbreak may have been; the resulting ‘likelihood’ map is also shown in figure 1*b*. From the MCMC output, there is a greater frequency of the initial infection being in either the northeast, the east or the southwest coastlines, with much higher likelihood in the northeast. It seems probable based on this evidence that the AFB infection originated in this area of Jersey. The specific cause cannot, of course, be ascertained from the dataset, although probable factors include, for example, the import of infected honeybees or equipment.

Infection times are resampled during the MCMC, and the order of infection for hives (including occult hives) are derived by the scheme. We can also use the changing infectious pressure throughout the epidemic to calculate the most probable source of infection for each infected hive, and whether the infection was more likely to be via the owner or by distance (by calculating the two terms in (2.2) and picking the larger). The one exception to this is the initial infection, which from our model set-up must be infected by random background transmission of AFB. One such example of the likely spread of infection of AFB from the MCMC scheme is shown in figure 1.

AFB generally seems to enter the island from the East, with more transmission events occurring by distance rather than the owner network. The length of jumps varies quite dramatically; most are less than 2 km, although there are rare instances where over half of the island is covered in a single transmission event. In the example in figure 1*c*, the majority of transmission events are due to the owner transmitting the disease (46 infections, compared to 40 by distance). Most of the longer range transmissions are caused by owners, although there are several infection events by distance that cover a large portion of the island.

The potential transmission rate of the average hive in the MCMC scheme was found to be approximately 0.02 hives per day. We stress that this is *not* an estimate of *R*_0_—as honeybee colonies are not observed to recover from AFB, a direct calculation of *R*_0_ from the dataset is not possible.

The number of occults present stays relatively low throughout the MCMC scheme. There are an average of around four undetected infections by the end of the inspection period, out of a possible 458 colonies.

### 3.3. Simulating epidemics

The dataset was generated by inspecting all hives on the island in the month of June (i.e. a complete census), and burning any hives that were found to be AFB-positive. This method of culling is the surest way to remove AFB from infected apiaries, but it is still not clear whether alternative measures could be used with increased efficacy in limiting an epidemic in honeybee populations. Using simulations, we test alternative methods of dealing with diseased hives, to find which is the most suitable for dealing with AFB. For each of the following methods, we use the spatial SIR model, begin with an infection at one hive (the hive and infection time are sampled from the MCMC output), and allow the disease to spread while imposing whatever control measures we choose. For all control strategies, we follow the same basic actions as the bee inspectors of Jersey did in 2010. For each control strategy, we run 10 000 replicates of the SIR model, and then investigate the resulting epidemics.

#### 3.3.1. Standard control practices

**Figure 2.**
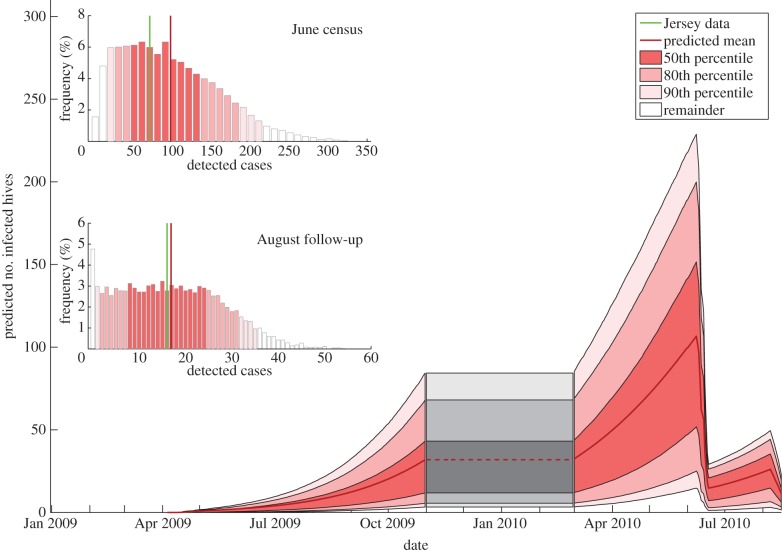
The size of epidemics when standard control strategies are taken. Shown is the mean behaviour, along with the 50th, 80th and 90th percentiles of the simulation data. The grey area represents the winter 2009/2010 ‘freeze’ period, where the number of infections is fixed and no disease transmission occurs. Over 10^4^ runs, approximately 3% of epidemics were eradicated by the control strategies.

Recreating the actions that bee inspectors took in Jersey (i.e. burning of infected hives, and secondary inspections of infected apiaries in August) results in the range of epidemics displayed in figure 2.

As seen in the two histograms, the mean sizes of epidemics, at the end of both the primary and follow-up inspections, are very similar to those observed in the dataset. This observation increases confidence that our model represents the true spread of disease, and the reliability of the parameter estimations in figure 6. This corroboration of a likelihood scheme to determine parameter values is not often observed in the literature, and we consider it important in backing up the choices made when constructing the mathematical model for disease spread. Another striking feature of the graph is that the epidemic is only very rarely stamped out by the inspection process. In the vast majority of cases, the June census removes only a portion of the infected hives, and then numbers begin to rise again during the (uninspected) period between the end of June and August, and the follow-up inspections are usually insufficient to eradicate the disease (with an average of around 11 undetected AFB-positive hives by the end of the August inspections). Thus, it is predicted that AFB was still present at the end of the inspection period in August 2010. This is corroborated by beekeepers' reports of AFB-infected hives the following year and again in 2012.

#### 3.3.2. Radial inspections

A common practice when dealing with infectious diseases is to cull all farms, regardless of infection status, within a certain radius of any infected animals discovered (contiguous premise culling, see [22]). The logic is that, if the disease spreads via local transmission, then by eradicating all animals within a certain distance of any cases, the chances of the disease spreading further are reduced. The effectiveness of this strategy is highly dependent upon the pathogen in question, and the method of transfer from animal to animal.

**Figure 3.**
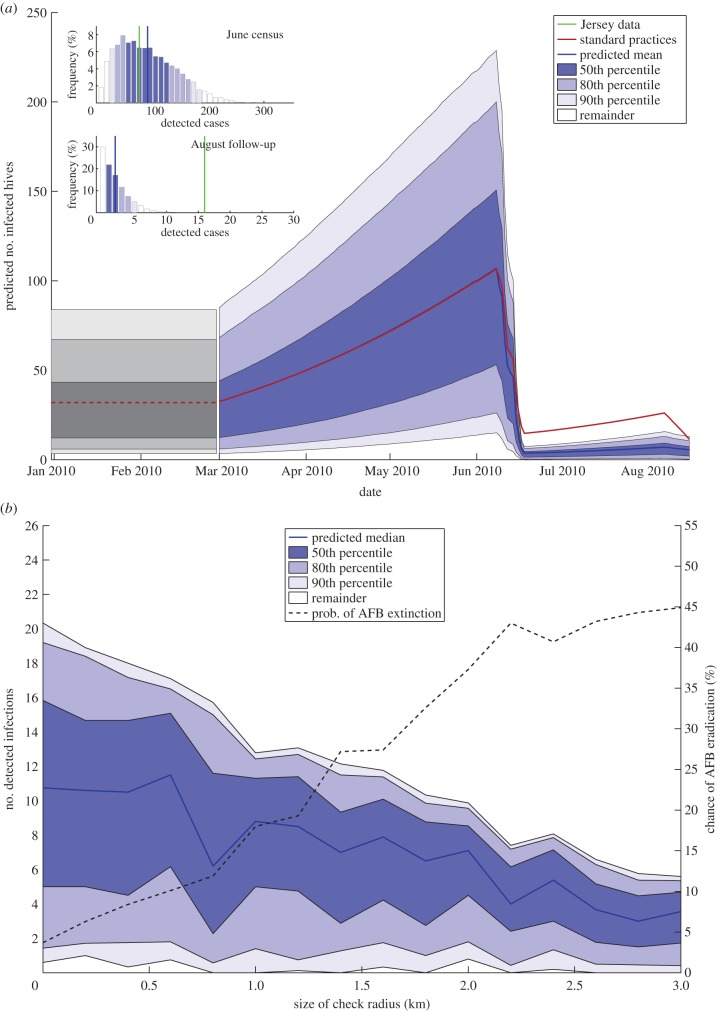
The effect upon epidemics when, after a case is detected, all hives within a certain radius radius are also checked. (*a*) The size of epidemics when a 0.5 km checking radius is used. Shown is the average behaviour, along with the 50th, 80th and 90th percentiles of the simulation data. Also shown is the average behaviour of the model without secondary inspections (red line). Note that in the histograms, it is the number of *primary* detections that are included—secondary detections are not counted so that the numbers can be directly compared to the original data. (*b*) The number of detected infections during the inspection period when secondary inspections are carried out at different sized radii. Shown is the average behaviour, along with the 50th, 80th and 90th percentiles of the simulation data. Also plotted is the probability of disease extinction at different radii (dashed black line).

A course of action which could be taken with regards to honeybee diseases is to *check* all apiaries within a certain radius of any AFB-positive hives found via inspection. Inspections take place on the same day, in order to reduce transmission as much as possible. We refer to these extra checks as *secondary inspections*. If AFB is discovered with these extra checks, then those hives are also destroyed, but otherwise the hives are not burned, and assumed to be susceptible. Figure 3 shows the size of epidemics when using such a method for secondary inspections.

The average behaviour of an epidemic is similar until the start of inspections (as control measures are yet to be applied), at which point the average decreases dramatically; by the end of June, the average number of infected hives is less than half of the value when secondary inspections are not carried out. By the end of the inspection period, approximately 90% of simulations result in smaller epidemics than without secondary inspections on average, as shown in figure 3*a*.

An obvious question when following this method of inspection is what radius is required to make a significant impact on disease prevalence. Invariably, the amount of manpower available will restrict how large a radius of secondary inspections are possible. Figure 3*b* shows the resulting epidemics from using different sized inspection radii, along with the probability that AFB is wiped out for each radius. As may be expected, the larger a checking radius that is used, the more disease is removed by inspections. Both the average and variance of the size of epidemics decrease as the size of the radius increases. The probability of wiping out AFB also increases from around 3 to 48% with a 3 km radius. It is intuitive that the more inspections that are carried out, the more infected hives will be detected and burned to prevent further disease spread; what is less obvious is how large to make this radius for secondary inspections. Unfortunately, detailed data on the costs of inspections were not available to us, so a detailed cost–benefit analysis is beyond the scope of this paper. However, it is worth noting that the entire area of Jersey is only 120 km^2^, so radii larger than 3 km would result in large portions of the island being inspected following detection of an AFB-positive hive (for example, if the radius was increased to 5 km, each single radial sweep around an infected hive would cover 78.5 km^2^, approximately 65% of the whole island).

#### 3.3.3. Earlier inspections

**Figure 4.**
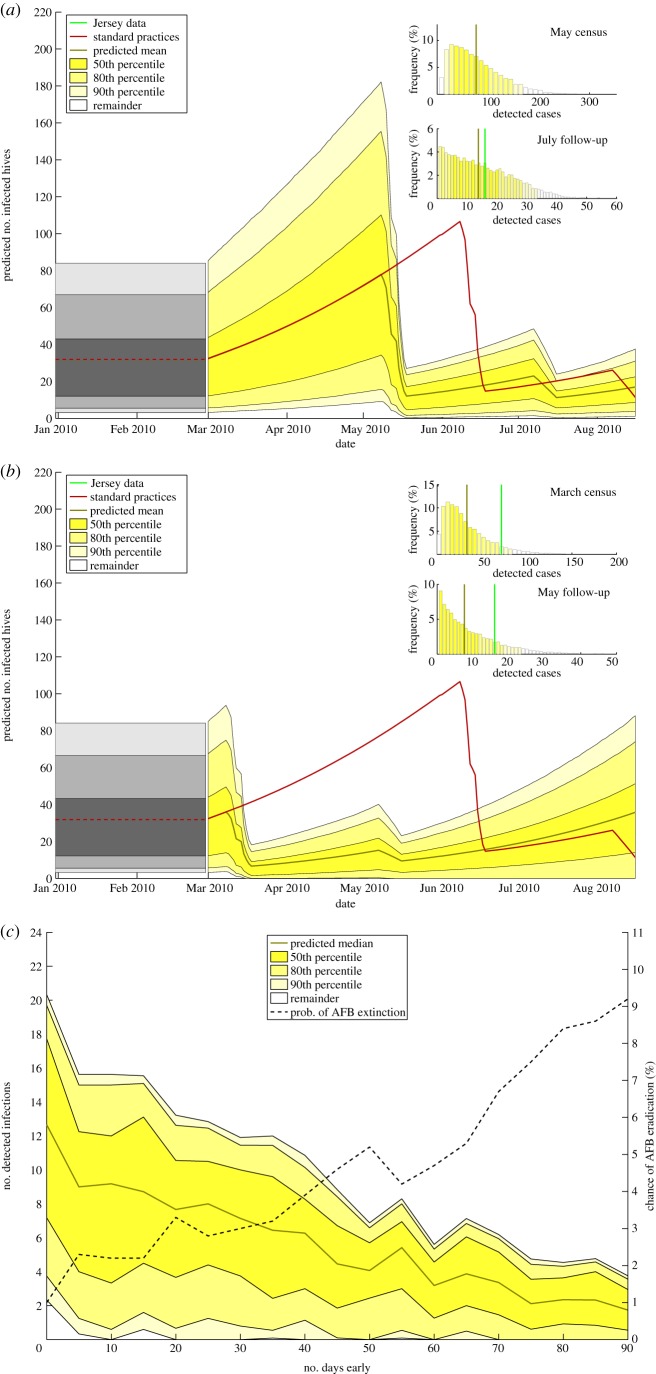
The effect on epidemics when all inspections are conducted earlier than in the original dataset. (*a*) All inspections moved 31 days earlier, i.e. the initial census was performed in the time period 9–18 May 2010, and the follow-up inspections in the period 8–16 July; note that in the histogram of the May census the data and predicted mean are extremely close, hence only one distinct line is visible. (*b*) All inspections moved 92 days earlier, so initial census performed in the time period 9–18 March 2010, and the follow-up inspections in the period 8–16 May. Shown is the average behaviour, along with the 50th, 80th and 90th percentiles of the simulation data. Also shown is the average behaviour of the model with inspections at the normal times (redline). (*c*) The number of detected infections during the inspection period when the timing of inspections are made earlier. Shown is the average behaviour, along with the 50th, 80th and 90th percentiles of the simulation data. Also plotted is the likelihood of disease extinction (dashed black line).

The number of infections during an epidemic tends to rise nonlinearly; owing to the initial geometric growth phase of a typical epidemic, a commonly posed question is how much the size of the epidemic could have been reduced if the initial detection had been earlier. If the initial census had been performed at an earlier stage, in theory fewer hives would be infected, and so the spread of disease would be more likely to be reduced. The results of allowing inspections to be earlier is shown in figure 4.

As expected, earlier inspections lead to the initial dip in the number of infected hives occurring earlier—during May in figure 4*a*, and during March in figure 4*b*. In March, after the end of the 2009–2010 winter freeze period, the number of infected hives is much lower, as AFB has not had the three extra months to spread before the original June inspections; thus, the drop in the number of infected hives is less pronounced than with standard practices (red line). As can be seen in the June histograms for both plots, there are on average fewer AFB-positive hives found by the end of the primary census than were observed during the actual epidemic (68.8 positive cases in May and 32.2 in March, compared to 70 from the data).

Interestingly, in neither case is the epidemic wiped out the majority of the time; the lower limit for the 50th percentile never reaches zero, although it is much lower for the March inspections than for the May ones. Thus, it seems the epidemic is more likely to be wiped out by performing the census earlier in time. This is confirmed in figure 4*c*, where the numbers detected, as well as the percentage likelihood of epidemic extinction, are plotted against how early the inspections are. Increasing the time that all inspections are rewound by has the effect of decreasing the number of detected AFB cases in the inspection period. This is because the epidemic is caught at an earlier stage, so fewer hives have been infected by the inspection dates. Because of this, the chance of eradicating the disease completely also increases, shown by the increasing chance of AFB eradication in figure 4*c*. The line is not monotonically increasing; owing to the stochastic nature of the SIR model, there is some variation in the chance of extinction. When the primary inspections are carried out 90 days earlier, the average chance of infection is around 9%, which is roughly equivalent to that when a 0.3 km secondary inspection radius is used (figure 3*b*).

### 3.4. Comparing control strategies

**Figure 5.**
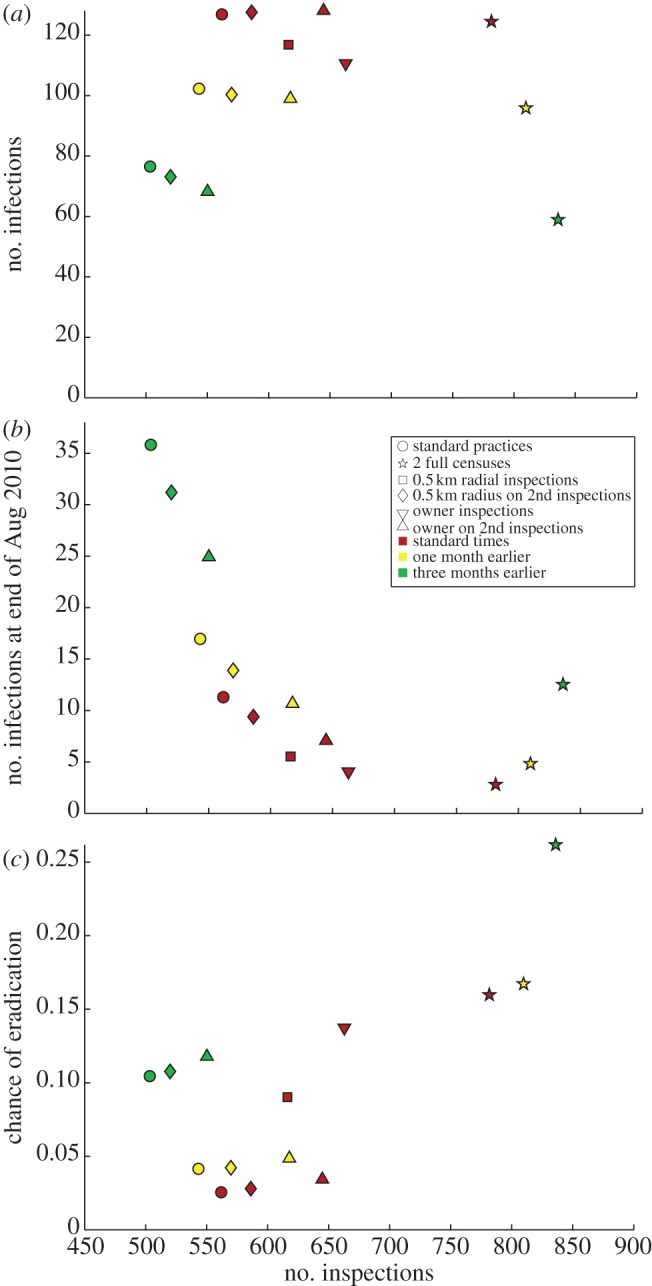
The comparison of different control strategies. Three different proxies are shown, all plotted against the number of inspections carried out; all values shown are means. (*a*) The size of epidemics. (*b*) The number of infections remaining on 16 August 2010, the last day for inspections in the original dataset. (*c*) The probability of wiping out AFB during the course of the inspections. Colours represent timings of the inspections, and shapes indicate the type of control strategy implemented (e.g. red circles show the results of following standard practices as employed on Jersey). Ninety-five per cent confidence intervals are too small to display on plots.

The previous two sections showed in detail the results of running two different control strategies. A whole suite of strategies were implemented, and the overall results comparing the different schemes are shown in figure 5.

Depending upon the desired result of the control strategy or the expense of carrying out inspections, different strategies would be optimal. Generally, the number of infections decrease the earlier the primary (and follow-up) inspections are carried out. This is indicated by the green points in figure 5*a*, representing initial inspections in March, being lower than the yellow points (May), which in turn are lower than inspections at normal times (June). Apart from inspections involving a follow-up census, all strategies result in 500–670 visits; employing a follow-up census pushes the number of inspections up to 780–850 inspections. Radial checks require fewer inspections than owner inspections, although all scenarios are within 100 inspections. For standard timing, it is better to do more inspecting early (e.g. radial checks for all inspections rather than just at the follow-up inspections), as more of the epidemic is stamped out early on. Note that in the case of two censuses, fewer inspections are carried out if inspections are carried out later in the year; this is caused by more hives being burned during the initial census (due to the epidemic being at a later stage), so that there are fewer hives to check during the follow-up inspections.

Conversely to figure 5*a*, earlier inspections result in a highest number of infections remaining at the end of August (figure 5*b*). This is due to the extra one or three months that any infected hives remaining have after the follow-up inspections in May/July, to spread disease unchecked to other hives during the remaining time. Thus, figure 5*b* may give an unrealistic view on the effectiveness of control strategy if observed on its own; we also need to look at the probability of wiping out AFB using the strategies (figure 5*c*). This shows a very different trend: making the primary inspections three months earlier with two complete censuses results in an extinction likelihood of around 26%, compared to only 2.5% using standard strategies. All other strategies result in an extinction likelihood of 10% or less, so if wiping out the disease is imperative, two censuses are required. Generally, for each control strategy type, the earlier the inspections begin, the better for controlling the size of the epidemic, as shown by the regular pattern of increasing chance of extinction in figure 5*c*.

## 4. Conclusion

Our starting point for the analysis carried out here was a dataset detailing the outbreak of AFB on Jersey during the summer of 2010. A census in June was proceeded by follow-up inspections in August, effectively providing two ‘snapshots’ of the epidemic, from which we attempted to reconstruct the entire epidemic. Such reconstructions are common for livestock, where generally data are more widely available [22,23,29], but are less common for honeybees. Using a Bayesian framework, an MCMC scheme was constructed to calculate both the parameter constants and infection times (of both known and unknown ‘occult’ infections, see Jewell *et al.* [20,21]) of a spatial SIR model with an underlying owner network, which we predicted would account for the majority of infection spread. We then used derived parameter values from the MCMC scheme to simulate epidemics, resulting in similar-sized epidemics (on average) as the data implied over the same time period (figure 2). We then simulated the consequences of implementing different control strategies in addition to the standard strategy (of burning infected hives and visiting the apiary two months later to confirm its AFB-negative status), to see what the best actions would have been, to reduce the size of the epidemics and/or increase the chances of wiping out the disease.

**Figure 6.**
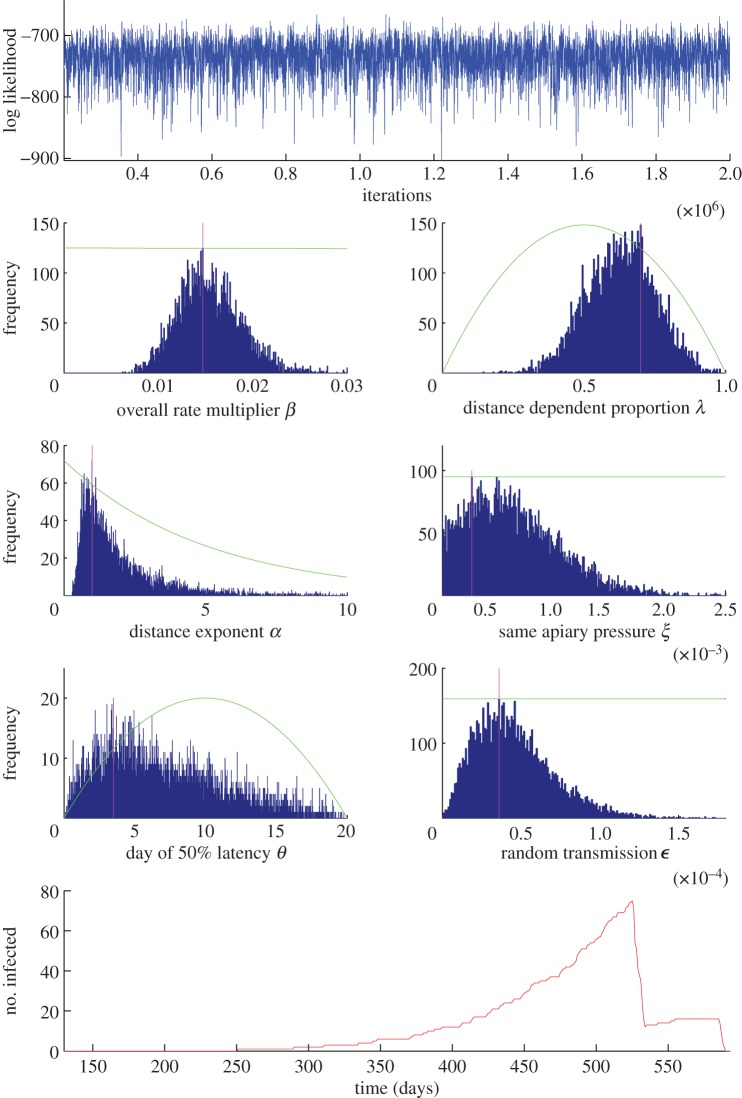
Results of the MCMC scheme, run for 1.8 × 10^6^ iterations (ignoring a burn-in period of 2 × 10^5^ steps). The vertical magenta lines indicate the modal values for the model constants. Also plotted are the prior distributions for the parameters (green lines). Plots are as follows: log likelihood, **β** (mode 1.47 × 10^−2^), *λ* (mode 0.70), **α** (mode 0.99), **ξ** (mode 2.6 × 10^−4^), **θ** (mode 3.49), **ε** (mode 3.6 × 10^−5^), numbers of current infected hives over time for one random run of the MCMC.

The mathematical model we built from the dataset is shown in §2.2. The results of the MCMC, with likelihood values calculated from (2.6), are summarized in figure 6 and show several key results. Both distance and ownership contributed significantly to the spread of AFB during the epidemic (shown by the distribution of **λ** in figure 6). This is confirmed by a sample run of the MCMC, where the most probable spread of infection is shown (figure 1*c*). Just over half of the infection spread was attributed to owner transmission in that instance, with the remaining spread due to distance. Long-distance transmission of AFB via the owner has been shown in the past [30], and our results corroborate this. The analysis also reveals the probability of the epidemic origin on the island, shown in figure 1*b*, to be in the northeast, or with a lower likelihood, in the east or the southwest of the island. This information could be key in determining how exactly the epidemic began—if one of the major sources of bee or equipment imports happens to be in the area, then the evidence heavily suggests that the disease entered the island via this method. Thus, measures could be taken to prevent future epidemics.

We constructed a stochastic SIR model to attempt to recreate the epidemic, using appropriate information from the data. The results were shown to correlate well with the data (figure 2)—the mean numbers of detected infections, in both June and August, were very close to the Jersey data, which shows that the model is a good indicator for the actual transmission process. We consider the forward simulation of epidemics using the results from the MCMC a key step in proving the reliability of the derived parameter values. The range of epidemic sizes over 10^4^ runs is, however, quite large, and the size of the actual epidemic (i.e. number of *infected* hives, not just *detected* hives) is likely to be significantly larger than the number of confirmed cases. We thus predict from our results that the disease was present after the end of the August inspections, and this hypothesis is backed up by several reports of AFB on Jersey the following year.

The control strategies we implemented include secondary radial checks and earlier inspections. In both cases, the measures were found to reduce the size of the epidemics and make disease extinction more likely. In the case of radial inspections, carrying out secondary inspections within 3 km of any confirmed cases resulted in an extinction probability of approximately 45% (figure 3*b*); however, the extra manpower involved to test so many extra hives may make this strategy prohibitive, and there will be a limit to how large a check can be carried out in the vicinity of AFB-positive hives. Unfortunately, data on the cost of AFB inspections were not available to us, so a rigorous cost–benefit analysis for secondary inspections is beyond the scope of this work.

Carrying out inspections earlier decreased the size of the epidemic by limiting the amount of initial spread before inspections began (figure 4*a,b*). Earlier inspections were also found to make disease extinction more likely (figure 4*c*); however, the increase over the three-month period that we examined did not lead to as large an increase in the likelihood of complete AFB removal as the radial inspections; primary inspections in June gave a 3% chance of disease extinction, whereas moving them back to March increased the probability to 8%. This probability is still relatively low, so further steps would be required to eradicate AFB entirely.

When comparing control strategies, results were mixed. Given that the first census occurred in June, all control strategies resulted in similar-sized epidemics (figure 5*a*), and the actions taken by Jersey bee inspectors resulted in fewer inspections than any further control strategies. Hence, if the cost of inspections is a limiting factor, the control measures taken were appropriate. In terms of limiting the spread of infection, the earlier the epidemic is discovered and action is taken, the smaller the resulting epidemic is (figure 5*a*). However, in practice with epidemics this is not always possible; depending on the disease, it may or may not be easily spotted by farmers, and by the time action is taken the epidemic may have already taken off. If wiping out the disease is the main aim, then two censuses are required to increase the chance of wiping out AFB (figure 5*c*). However, the number of inspections required to carry out this strategy is much higher, and the costs may be too prohibitive for such action to be taken. We have provided a general framework here which can be used, in conjunction with economic data about inspections costs, to provide an optimum strategy to follow for future epidemics.

Other control measures not carried out in our simulations include shook swarm methods [17] and the use of OTC as an antibiotic against AFB and EFB [7,17]. There is no unified approach to the control of honeybee diseases; for example, only recently, experimental work has shown the benefits of shook swarm over OTC-based measures [19], and measures differ between countries (for comparisons between the USA, the UK and New Zealand, see [15,16,31]). OTC resistance has been observed in recent experiments [32,33], and alternative measures to antibiotics have been explored such as breeding bees for an increased immune response to AFB [34] and natural alternatives to antibiotics [35,36]. The reason we chose to avoid simulating extra control measures is a lack of quantitative data about the effectiveness of shook swarm and OTC. With more specific data, such control measures would not be difficult to implement computationally. Depending upon the performance of such control measures, smaller epidemics may result in the future.

This is the first rigorous statistical analysis carried out on a honeybee disease epidemic that we are aware of, and several issues were found. First of all, the methods of disease transmission that we accounted for included: distance, owner, within-apiary and random (background) transmission. A more rigorous model would include other links to facilitate disease transmission, such as apiarists sharing equipment and hive movement between apiaries. Unfortunately, this required much higher resolution data than we possessed; livestock movement data are usually well documented (see [22,37]), and in our model, we assumed all hives stay in the same apiary for the period of the simulation. Information about imports of bees (which is controlled by legislation on Jersey, and a licence required for the import of queens) would no doubt be useful in determining the likely origin of epidemics, especially for an island such as Jersey where bees are unlikely to travel from other locations (although one beekeeper did report seeing a swarm travelling mid-Channel between Jersey and France, highlighting the possibility of honeybee influxes from mainland Europe).

Nevertheless, as a starting point, we believe that our analysis shows great potential in helping to limit future epidemics in honeybees. We have established clear links between both proximity and ownership and the spread of AFB, and shown the speed at which the epidemic probably grew. There is a very high probability that the disease was present from the previous year, but at low enough numbers to go unnoticed. We have also shown how control measures can be used to minimize the size of the overall epidemic. In the future, we hope to use the statistical framework established in this analysis to investigate the spread of EFB in England and Wales, using data available since 1993. It is hoped that a much larger dataset will enable us to provide more robust conclusions, and comparisons between the spread of EFB and AFB could potentially lead to different control strategies needed to reduced the size of epidemics. Finally, the fact that all these findings can be revealed from two spatial snapshots of the infection status suggests that these techniques can be applied to a wide range of outbreak scenarios without the need for costly high-resolution temporal data.

## Figures and Tables

**Figure 7.**
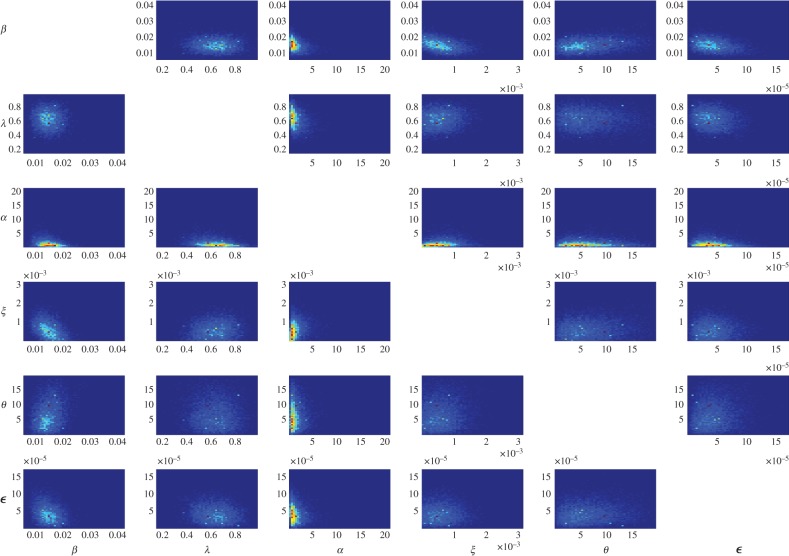
Plotting model constants against each other. Note that although upper and lower diagonal plots are symmetric, axis scaling can differ, leading to variations in the shapes of scatter plots.

**Figure 8.**
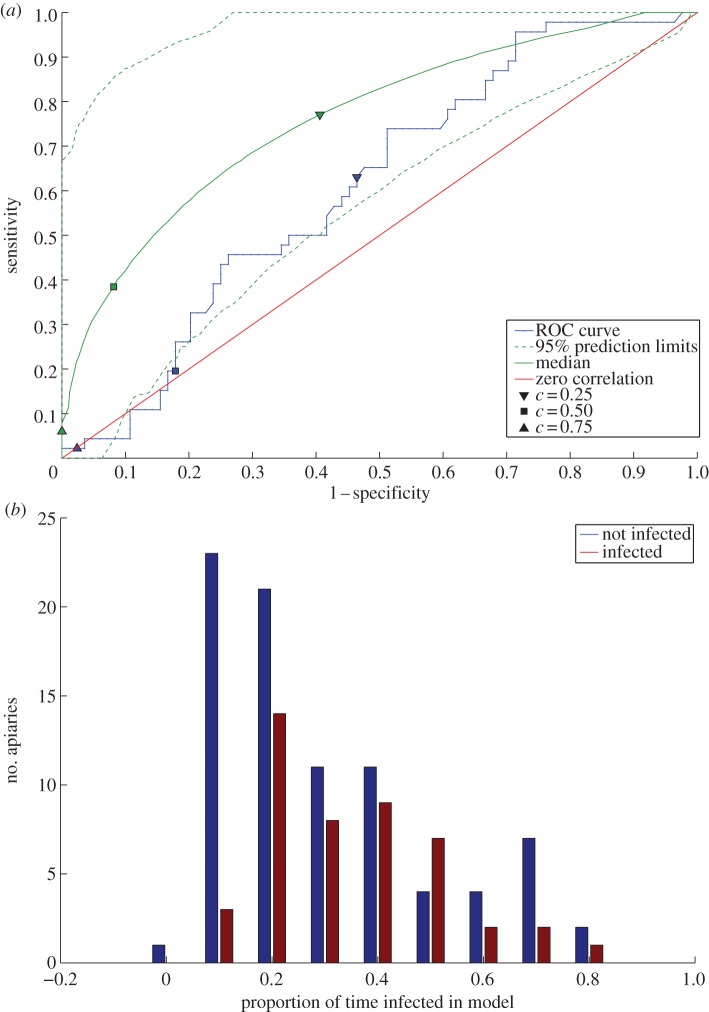
Summary of comparing the model results to the data. (*a*) ROC curves showing the sensitivity of the model results to the data (solid blue line). Also shown are the zero correlation line (dashed-dotted red line), the 95% prediction limits from the model (dashed green lines) and median from the model (solid green line). Marked on both the model–data comparison and model median lines are the locations of *c* = 0.25, *c* = 0.5 and *c* = 0.75. (*b*) Bar chart showing a comparison of the proportion of times an apiary is infected in the model, dependent on whether it is infected in the data for the June census.

## Appendix A

### A.1. Model construction

The overall aim of any MCMC scheme is to try to reconstruct the series of events which took place, leading to the dataset possessed at present. For this, an appropriate model which captures the behaviour of the system accurately is paramount for good results. In the case of epidemic data, it is thus important to capture the main methods for the spread of the disease, in order to have a high chance of recreating the epidemic. As we have information about both the geographical location and owner of each hive, it makes sense to use this information in construction of the function for disease spread. Owing to a lack of knowledge about the relative strengths of distance and ownership in the transmission of disease between hives, we set up our transmission function as

A1

In this way, 0 ≤ λ ≤ 1 shows the relative amount of spread due to the two factors—the closer to 1 that **λ** is, the more important the proximity between hives is for infection spread. We include **ξ** as an extra pressure which applies between hives on the same apiary—this is due to extra interactions which may occur at the within-apiary level, such as bees travelling between neighbouring hives, acting as a vector for AFB. With the inclusion of disease latency and the background transmissions of disease, the infectious pressure upon any hive is thus given by (2.4).

The likelihood function for our epidemic has to take the following probabilities into account:

— susceptible hives staying uninfected, either for the entire inspection period (in which case we set **I**(*j*) = *T* + 1) or from the beginning of the inspection period until **I**(*j*) (if **I**(*j*) ≤ *T*);
— susceptible hives becoming infected at time *t*, under infectious pressure from other hives or random infection;
— infected hives being detected when an inspection takes place; and
— infected hives not being detected when an inspection takes place (i.e. false negatives).

We model the inspection period as a discrete-time process, with each step being 1 day; this is logical, as the inspection data are categorized by the date each inspection occurred. In discrete time, we convert our infectious pressure (2.4) into a probability of infection, which is a Poisson process with probability

A2

For a detailed method of dealing with discrete-time models, see [38]. The likelihood calculation is thus

A3

Here, the first term is the probability of *not* being infected on day *t*, which is  (infection) from (A 2), while the second term follows (A 2) exactly; both terms involve summing over all hives. The third and fourth terms correspond, respectively, to AFB-positive hives being detected and not detected, following the detection function (2.5).

Initially, owner compliance was included in the model, which allows for owner-based transmission to be reduced once inspectors were made aware of the presence of AFB and alerted farmers. However, it was not significant to the model results (i.e. there was no information gained from the likelihood scheme) and thus was not included. We hypothesize that the nature of the data collection (i.e. returning only to infected apiaries in August rather than a second complete census of all hives) provided insufficient information to test for owner compliance.

### A.2. Prior distributions

To set up the MCMC scheme, we must first set initial values for all our parameters; the job of the MCMC algorithm will then be to calibrate these parameters, and the output tends to the most probable system configuration. For our model, the unknowns are the constants in the model (**β**, **λ**, **α**, **ξ**, **θ**, **ε**) and the infectious periods for all confirmed infected hives. As well as this, we attempt to locate any occult infections which were not detected during the inspection period.

In the case of the constants, values must be positive. For parameters with no obvious upper limit, we choose priors to be gamma distributions,

A4

By definition **λ** needs to be in the range [0,1] so we use a beta distribution as our prior,

A5

centred at 0.5. With information from FERA and the bee inspection team from Jersey, we assume an upper limit of 20 days for the latency period. Thus, we use a modified beta distribution as our prior for **θ**,

A6

For setting infectious periods, we opt for either shifting infection times by 1 day either side or picking random values from an exponential distribution (see §A.3).

### A.3. MCMC Algorithm

We are now ready to run the MCMC scheme. As likelihood values tend to be very low, it is beneficial (for machine accuracy) to work with log likelihoods, henceforth denoted by *L*. The initial likelihood is set extremely low, *L*_old_ = −10^6^. There are five possibilities for the adjustment of parameters in our scheme, and one adjustment is chosen at each iteration. In general, whatever step is taken, we calculate the new likelihood after the change (*L*_new_), before calculating the following value:

A7

where *Q* is the proposal distribution (e.g. [39]). Generally, *Q* is made up of two components: the probability of picking the old value given the new value, and the probability of picking the new value given the old value. Together, these give

A8

where *q*(*x*) is the prior distribution for the parameter *x* (this can be either a model constant or an infection time).

The new set of parameters is accepted if *A* > *u*, where *u* ∼ *U* [0,1]; in other words, the new parameters are definitely accepted if *A* > 0, and with probability e*^A^* if *A* < 0. The method of updating values, along with the calculation of the value of *Q* is as follows for the five different parameter changes:

(1) Varying the value of one of the model constants. We choose one of the model constants at random, and move a small amount from the old value of the constant. We sample from a normal distribution and add this value to the old parameter value
  A9
  where the variance **σ** is proportional to the initial value of the constant; initial values used are estimates of the constants from preliminary runs of the MCMC, so constants with higher initial values have higher variances. As we are using a symmetrical distribution to calculate the new value, the first fraction of (A 8) is 1.
(2) Varying the infection time of some of the infected hives. This involves first picking a random number of infected hives to resample infection times for. Secondly, we resample infection times for each hive; for this we either move the infection time either side by 1 day, or sample from an exponential distribution
  A10
  For early iterations, infection times are often generated by moving 1 day to either side of the old time, so that we explore the parameter space of infection times thoroughly. As the number iteration increases, times are resampled more frequently from the exponential distribution (A 10). We set *γ* = 200 days for sampling infection times from, to allow a wide range of infectious periods. We also impose an upper limit of 300 days, such that infectious periods are resampled if a period greater than 300 days is calculated.
(3) Introducing an infection to one of the susceptible hives, and setting its infection time (known as an occult infection). A susceptible hive is selected at random to become infected, and the infectious period *T −* **I**(*j*) is sampled from a uniform distribution
  A11
  This is subtracted from the end of the inspection period *T* to give the infection time. We use a uniform distribution instead of the exponential distribution (A 10) for occult infections, as an occult is extremely unlikely to be infected long before the inspection date; we assume a 30-day limit before the inspection. The value of *Q* for a new infection is adapted from Jewell *et al.* [20,21] and is defined as
  A12
  where *O* is the number of occults before the current addition.
(4) Removing one of the occult infections. The value of *Q* is again adapted from Jewell *et al.* [20,21] and is defined as
  A13
(5) Varying the addition or the removal time of one of the hives which is not present for the entire inspection period (as the numbers of hives in apiaries sometimes varied between inspections without explanation). As the lower and upper limits for the removal/addition time are fixed (as the two inspection dates  and ), we simply use a uniform distribution
  A14
  where the time of the change *C*(*j*) must be after the first inspection day and before the second. As we are sampling from a uniform distribution, *Q* = 0.

The parameter change that we choose is selected from the above five options such that infection times are resampled with a higher frequency at the beginning of the MCMC scheme. Thus by the time we start to vary other parameters, we are more likely to be in the right region of parameter space for the infection times of diseased hives.

### A.4. MCMC output

For the MCMC scheme to have explored the multi-dimensional parameter space thoroughly, we allow the scheme to run for 1.8 × 10^6^ iterations, including a burn-in period of 2 × 10^5^ steps where values are not recorded. The results are shown in figure 6.

The plot of the log-likelihood varies throughout the iterations, as expected in an MCMC scheme, with the values distributed roughly around log(*L*) = −800. The shape confirms that the parameter space is being explored thoroughly, with an acceptance of 56.3%. This acceptance rate is higher than the ideal acceptance range of 16–40%, although we highlight that the quoted range is applicable when only step 1 of the MCMC scheme is carried out (i.e. altering parameter constants, see §5), and the acceptance rate for this step alone is 29.2%. The histograms for the model constants mostly have Gaussian shapes, with the exception of 50% latency day, which is more evenly spread over the 20-day period. The histogram for **λ** is centred around 0.6, meaning that just over half of all transmission is due to distance-dependent transmission rather than by owner. Thus, both pathways are significant in controlling the spread of AFB.

To test for parameter independence, we plot the model constants against each other, producing a set of cloud plots (figure 7).

### A.5. ROC curves

To test the reliability of the SIR model used to simulate epidemics, we plot ROC curves to test the sensitivity and specificity of the model compared to the data, along with a histogram comparing the infection status of the model and data (figure 8).

The ROC curve is formulated by defining a cut-off *c* between 0 and 1; apiaries are determined to be positive, if they are infected in more than a proportion *c* of simulations and negative otherwise. The ROC curve is then drawn by varying the value of *c*. For all *c* > 0.5, our ROC curve lies above the diagonal line, showing that our simulations have better than random predictive accuracy.

The constants appear to be independent, with little covariance between them (i.e. positive and/or negative correlations). This corroborates our model formulation.

However, our modelling methodology is not a simple statistical fit to the data (in the traditional sense), but aims to generate the underlying mechanistic processes driving transmission. For this reason, simple measures of agreement between predictions and data are not readily applicable, especially given the stochastic variation between simulations. To overcome this issue, we also use the ROC curve to assess how well the model performs at predicting a single model simulation (i.e. treating one simulation from the model as the true data), and use this comparison to generate mean and 95% prediction intervals of this ideal (green lines in figure 8*a*). This model–model comparison highlights the effects of stochasticity and provides an upper bound on what could be achieved even if the model perfectly captured the underlying mechanisms. We find that the ROC curve that compares model and data lies within the 95% prediction intervals of what can be expected for *c* > 0.5.

Finally in figure 8*b*, we separate apiaries that we found positive in the June census (red) from those found negative (blue). For each of these, we show frequency histograms of the proportion of simulations in which an apiary is infected. Clearly, apiaries positive in the June census are more likely to be infected in the model, while apiaries rarely infected in the model are generally ones where AFB was not detected.

The difficulty of forming a simple statistical comparison between model and data is complicated by two main factors: (i) the variability in model results due to both the stochastic nature of transmission but also the uncertainty in parameter values from the MCMC scheme; (ii) the fact that the observed epidemic is simply one realization of the possible epidemics that could occur, and there is no reason to believe that this was in any way a typical epidemic.
